# Ion Therapy: A Novel Strategy for Acute Myocardial Infarction

**DOI:** 10.1002/advs.201801260

**Published:** 2018-11-08

**Authors:** Min Yi, Hekai Li, Xiaoya Wang, Jianyun Yan, Long Gao, Yinyan He, Xinglong Zhong, Yanbin Cai, Weijing Feng, Zhanpeng Wen, Chengtie Wu, Caiwen Ou, Jiang Chang, Minsheng Chen

**Affiliations:** ^1^ Department of Cardiology Heart Center Zhujiang Hospital Southern Medical University Guangzhou Guangdong 510280 China; ^2^ State Key Laboratory of High Performance Ceramics and Superfine Microstructure Shanghai Institute of Ceramics Chinese Academy of Sciences 1295 Dingxi Road Shanghai 200050 China; ^3^ Guangdong Provincial Biomedical Engineering Technology Research Center for Cardiovascular Disease Guangzhou Guangdong 510280 China; ^4^ Sino‐Japanese Cooperation Platform for Translational Research in Heart Failure Guangzhou Guangdong 510280 China; ^5^ Institute for Stem Cell and Regeneration Chinese Academy of Sciences Beijing 100101 China

**Keywords:** acute myocardial infarction, bioactive ions, therapy

## Abstract

Although numerous therapies are widely applied clinically and stem cells and/or biomaterial based in situ implantations have achieved some effects, few of these have observed robust myocardial regeneration. The beneficial effects on cardiac function and structure are largely acting through paracrine signaling, which preserve the border‐zone around the infarction, reduce apoptosis, blunt adverse remodeling, and promote angiogenesis. Ionic extracts from biomaterials have been proven to stimulate paracrine effects and promote cell–cell communications. Here, the paracrine stimulatory function of bioactive ions derived from biomaterials is integrated into the clinical concept of administration and proposed “ion therapy” as a novel strategy for myocardial infarction. In vitro, silicon‐ enriched ion extracts significantly increase cardiomyocyte viability and promote cell–cell communications, thus stimulating vascular formation via a paracrine effect under glucose/oxygen deprived conditions. In vivo, by intravenous injection, the bioactive silicon ions act as “diplomats” and promote crosstalk in myocardial cells, stimulate angiogenesis, and improve cardiac function post‐myocardial infarction.

## 1 Introduction

Eliminating vascular obstruction and promoting angiogenesis can reduce myocardial apoptosis and necrosis, which is critical in the treatment of acute myocardial infarction (AMI).^1^ Despite the most advanced pharmacological and medical device treatment methods were used, neither of these promote cardiac angiogenesis and neovascularization nor stimulate the endogenous repair leading to steadily increasing incidence of heart failure as well as poor prognosis.^2^ Although some cell‐based or noncellular biomaterial based approaches and cotransplant cells^3^, ^4^ with synthetic biomaterials strategies are under trial, they are still facing great challenge for clinical applications. In particular, the poor retention and engraftment of transplanted cells as well as the difficulty in implantation and degradation of biomaterials discounts the therapeutic effect dramatically.^3^, ^5^ Recent studies have shown that one remarkable mode of therapeutic action is that the injected stem cells as well as its membrane function through the secretion of paracrine factors^6^, ^7^ and trigger intracellular protective/regenerative pathways in the host cells^8^, ^9^ to promote endogenous repair. These results indicate that stimulating the crosstalk among cardiac host cells and promoting endogenous angiogenesis are essential in re‐establishing blood supply flow and may rescue the existing working cardiomyocytes and improve the prognosis of patients post‐AMI. Therefore, development of strategies or substances to regulate cardiac host cells, in particular the interaction between endothelia cells and cardiac cells and enhance angiogenesis are critical for myocardial infarction.

Previous works including our studies have found that silicate‐based biomaterials, especially bioactive glasses (BGs) and the silicate ceramics (CS) can promote cell–cell communications by affecting gap junction associated Connexin 43 (Cx43) mediated endothelial cell behavior,^10^ improving the interactions between human umbilical vein endothelial cells (HUVECs) and bone marrow stromal cells (BMSCs) or human dermal fibroblasts (HDFs) and stimulating angiogenesis both in vitro and in vivo, and further enhance tissue regeneration.^11^, ^12^, ^13^ Moreover, the bioactivity of these silicate based biomaterials mainly rely on the released ions, in particular the silicon ions.^11^ Inspired by the functions of these bioactive materials and stem cell therapy, we proposed a novel therapeutic strategy—“ion therapy,” in which bioactive ions derived from silicate biomaterials are intravenously injected and act as “diplomates” to regulate cell–cell communications and angiogenesis. Our hypothesis is that the intravenously injected ions may on one side activate cardiac cells and on the other side stimulate angiogenesis, and finally inhibit cell necrosis and rebuilt the blood supply. In this study, the therapeutic effects of “ion therapy” on the endogenous myocardium repair and neovascularization were investigated, and we demonstrated for the first time, that the intravenously injected Si ions indeed have significant therapeutic effect on AMI by regulating cell behaviors and angiogenesis.

## 2 Results

### 2.1 Ion Concentrations of CS Extracts and Effects of Silicon‐Enriched Ion Extracts on Neonatal Rat Cardiomyocytes Cell Viability

**Figure 1:**
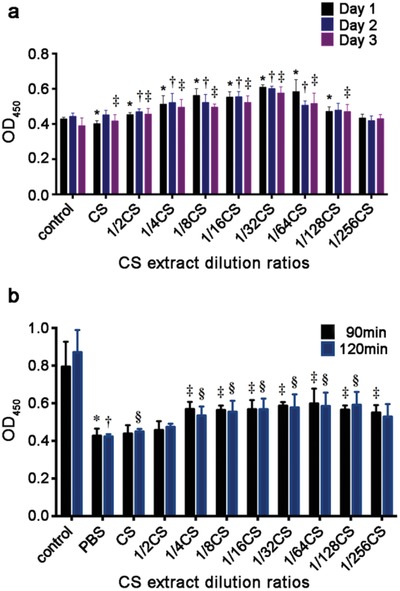
Effects of silicon‐enriched ion extracts on NRCMs cell viability under normal and glucose/oxygen deprived conditions. a) NRCM cell viability cultured in different CS extracts diluted with DMEM for 1, 3, and 5 d under normoxia conditions (*p < 0.01 vs control group for Day 1; †*p* < 0.01 vs control group for Day 3; ‡ *p* < 0.01 vs control group for Day 5; *n* = 6 for each group). b) NRCMs cell viability cultured in different CS extracts diluted with PBS for 90 and 120 min under glucose/oxygen deprived conditions. Control group was cultured in normal condition for an equivalent time (*p < 0.01 versus control group for 90 min; †*p* < 0.01 vs control group for 120 min; ‡*p* < 0.01 vs PBS group for 90 min; §*p* < 0.01 vs PBS group for 120 min; *n* = 6 for each group). All data are obtained from three independent experiments; mean ± SD.

To investigate the effects of bioactive ions on neonatal rat cardiomyocytes (NRCMs) cell viability, CS extracts and serial dilutions of extracts (1/2, 1/4, 1/8, 1/16, 1/32, 1/64, 1/128, and 1/256) were prepared by phosphate buffered solution (PBS) or Dulbecco's modified Eagle medium (DMEM) as previously described. The ion concentrations of Ca, P, and Si were determined by inductively coupled plasma atomic emission spectroscopy (ICP‐AES) and the results are shown in **Table** **1**. It is found that Ca and P ion concentrations in DMEM diluted CS extracts and 1/16CS were much lower than that of DMEM while Si ion concentrations in CS extracts and the dilutions at 1/16, 1/32, 1/64, 1/128, and 1/256 ratios were significantly higher than that of DMEM. In DMEM diluted CS at 1/32, 1/64, 1/128, and 1/256 ratios, no significant differences were found in Ca and P concentrations when compared to DMEM. Next, we investigate the ion concentrations of PBS diluted CS. No significant difference was found in Ca concentrations between all groups and P ion concentrations in PBS diluted CS extracts, 1/16CS and 1/32CS were much lower than that of PBS while no difference were found at 1/64, 1/128, and 1/256 ratios. Furthermore, Si ion concentrations at all diluted ratios were significantly higher than that of PBS. It is interesting to note that the Ca and P concentrations in original CS extracts in DMEM or PBS are not only clearly lower than that in DMEM or PBS, respectively, but also lower than that in the corresponding extracts dilutions. This is possibly due to the calcium phosphate precipitation in DMEM or PBS induced by silicates as reported previously.^14^, ^15^ Then, in order to evaluate the protective effect of Si ions, NRCMs cell viability under normoxia and glucose/oxygen deprived conditions were examined in the presence of silicon‐enriched ion extracts. **Figure** **1**a reveals that NRCMs cell viability cultured for up to 5 d in 1/8, 1/16, 1/32, and 1/64 CS extracts diluted with DMEM are higher than that of control medium under normoxia condition while no difference was observed between the original CS extract and the control suggesting a stimulatory effect of Si ions in certain concentration range on NRCMs under normal culture condition. In contrast, under glucose/oxygen deprivation condition in PBS, NRCM cell viability was apparently decreased when compared to normal condition. It is interesting to note that the viability of the cells cultured in CS extracts diluted at 1/8, 1/16, 1/32, and 1/64 CS ratio are higher than that in PBS and the highest cell viability is found at the concentrations between 1/32 and 1/64 ratios (Figure 1b). These observations indicated that glucose/oxygen deprivation harmed NRCMs and resulted in a decrease of the cell viability whereas silicon ions can alleviate this damage and have potential protective effect on NRCMs.

**Table 1:** Ion concentrations of CS extracts diluted with DMEM medium and PBS

	Ca [µg mL^−1^]	P [µg mL^−1^]	Si [µg mL^−1^]
DMEM	59.33 ± 0.97	25.22 ± 0.34	0.02 ± 0.01
(1)CS in DMEM	5.11 ± 0.25b)	6.37 ± 0.09b)	113.59 ± 0.30b)
CS/1/16 in(1)	56.19 ± 0.46a)	23.92 ± 0.38a)	6.66 ± 0.05b)
CS/1/32 in(1)	57.89 ± 0.62	24.98 ± 0.68	3.36 ± 0.04b)
CS/1/64 in(1)	58.92 ± 0.73	25.24 ± 0.70	1.63 ± 0.02b)
CS/1/128 in(1)	59.47 ± 0.42	25.01 ± 0.28	0.80 ± 0.02b)
CS/1/256 in(1)	59.62 ± 0.55	25.13 ± 0.52	0.41 ± 0.01b)
PBS	0.06 ± 0.00	274.44 ± 1.00	0.12 ± 0.01
(2)CS in PBS	0.05 ± 0.01	32.52 ± 0.44d)	283.88 ± 3.43d)
CS/1/16 in(2)	0.08 ± 0.01	260.22 ± 0.55d)	17.42 ± 0.37d)
CS/1/32 in(2)	0.06 ± 0.00	268.08 ± 0.63c)	9.08 ± 0.75d)
CS/1/64 in(2)	0.06 ± 0.00	270.91 ± 0.81	4.53 ± 0.18d)
CS/1/128 in(2)	0.05 ± 0.00	273.06 ± 0.10	2.26 ± 0.03d)
CS/1/256 in(2)	0.05 ± 0.00	273.23 ± 0.79	1.25 ± 0.02d)

^a)^ *p* < 0.05 and

^b)^ *p* < 0.001 when compared with concentration of ions in DMEM medium.

^c)^ *p* < 0.05 and

^d)^ *p* < 0.001 when compared with concentration of ions in PBS; All data are obtained from three independent experiments; mean ± SD.

### 2.2 Effects of Silicon‐Enriched Ion Extracts on Apoptosis of NRCMs under Glucose/Oxygen‐Deprived Conditions In Vitro

#### 2.2.1 Terminal Deoxynucleotidyl Transferase dUTP Nick End Labeling Staining of NRCMs under Glucose/Oxygen Deprived Conditions

**Figure 2:**
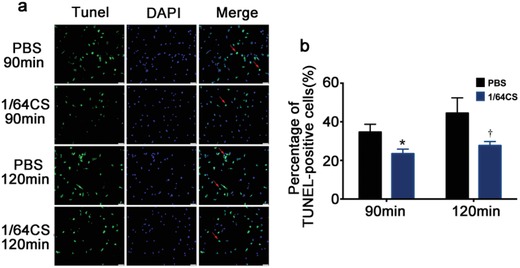
Effect of silicon‐enriched ion extract on apoptosis of NRCMs under glucose/oxygen deprived conditions in vitro. a) Representative pictures of NRCMs with TUNEL staining (green) and DAPI (blue) under glucose/oxygen deprived conditions in vitro. Red arrows show TUNEL‐positive NRCMs. Scale bar represents 50 µm and all data are obtained from three independent experiments. b) Quantitative analysis of TUNEL‐positive NRCMs (10 pictures for each group). *p < 0.05 versus PBS‐90 min, †*p* < 0.05 versus PBS‐120 min; mean ± SD.

In vitro, the glucose/oxygen deprivation induced NRCMs apoptosis was used to mimic the AMI condition in vivo. Cardiomyocytes apoptosis was investigated by TUNEL staining and the percentage of TUNEL positive cells were calculated. The results are shown in **Figure** **2**a,b. It is clear to see that under glucose/oxygen deprived conditions, NRCMs went into apoptosis after culturing for 90 min, and with the prolonged culture time up to 120 min, the apoptosis was clearly increased as seen in the increase of the numbers of TUNEL‐positive cells (Figure 2a). In contrast, a significant decrease of TUNEL‐positive cells was observed in the cells treated with 1/64 CS extract both after 90 and 120 min culturing indicating an inhibitory effect of the bioactive ions on NRCMs apoptosis (Figure 2a). Most interestingly, the inhibitory effect of Si ions was slightly stronger in 120 min group than that in 90 min group. This may be because the damage of the cells in glucose/oxygen deprived conditions for 90 min was much lower than that for 120 min so that the protective effect of silicon ion extracts was not that obvious. In addition, the effect of silicon‐enriched ion extracts on ROS level and proliferation of NRCMs under glucose/oxygen deprived conditions was also investigated. The results revealed that the silicon‐enriched ion extract clearly decreased the expression of ROS in NRCMs (Figure S4, Supporting Information).The gene expression of cTnT and Myh6 was increased in 1/64CS treated group in normal condition. However, under glucose/oxygen deprivation condition, only cTnT gene was significantly increased in 1/64CS group (Figure S2, Supporting Information).

#### 2.2.2 Expression of Apoptotic‐Associated Mitogen‐Activated Protein Kinase Family Proteins and Cleaved‐Caspase 3 in NRCMs under Glucose/Oxygen Deprived Conditions In Vitro

**Figure 3:**
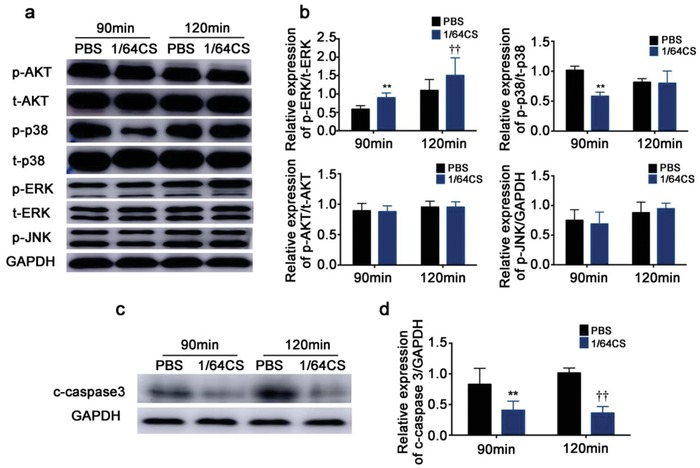
Effect of silicon‐enriched ion extract on the expression of apoptotic‐associated MAPK family proteins and cleaved‐caspase 3 in NRCMs under glucose/oxygen deprived conditions in vitro. a) MAPK family protein expression measured by western blot and GAPDH was served as the loading control. b) Quantification of bands by densitometry. c) Cleaved‐caspase 3 protein expression measured by western blot and GAPDH was served as the loading control. d) Quantification of bands by densitometry. Data are obtained from three independent experiments. **p < 0.01 versus PBS‐90 min, ††*p* < 0.01 versus PBS‐120 min; mean ± SD.

To study the underlying mechanism, we then determined mitogen‐activated protein kinase (MAPK) family proteins and cleaved‐caspase 3 protein expression in glucose/oxygen deprived induced apoptosis of NRCMs. It is known that p38 is involved in cell apoptosis and here we observed that silicon enriched 1/64CS extract affected p38 expression of NRCMs by inhibiting the phosphorylation of p38 (**Figure** **3**a,b), while the expression of nonphosphorylated p38 was not affected. The time and duration of p38 phosphorylation in different conditions varied. At 90 min, we found that p38 phosphorylation was clearly inhibited by 1/64CS extract, while the inhibition disappeared after culturing for 120 min. This is in consist with the in vivo result that the expression of phosphorylated P38 protein was also increased in the border area of infarcted myocardial (Figure S8, Supporting Information). We further found that ERK1/2, another important protein which is known to inhibit cell apoptosis, was also affected by 1/64CS extract. The expression of the phosphorylated ERK1/2 of NRCMs was significantly enhanced both after 90 and 120 min culturing. In contrast, the expression of p‐JNK, AKT, and p‐AKT was not affect by 1/64CS extract (Figure 3a,b). More interestingly, we found that the 1/64CS extract can also regulate the expression of another major protein of downstream apoptotic effector molecule, the cleaved caspase‐3 protein. As shown in Figure 3c,d, the expression of cleaved caspase‐3 expression of NRCMs was significantly downregulated as compared to that in PBS groups both after 90 and 120 min culturing. Generally, these findings provided evidences that silicon‐enriched ion extract may execute post‐traumatic cardiac protection through attenuating cardiomyocyte apoptosis by regulating apoptosis‐associated proteins expression.

### 2.3 Effect of Silicon‐Enriched Ion Extract on the Expression of Gap Junction Associated Cx43 in NRCMs under Glucose/Oxygen Deprived Conditions In Vitro

**Figure 4:**
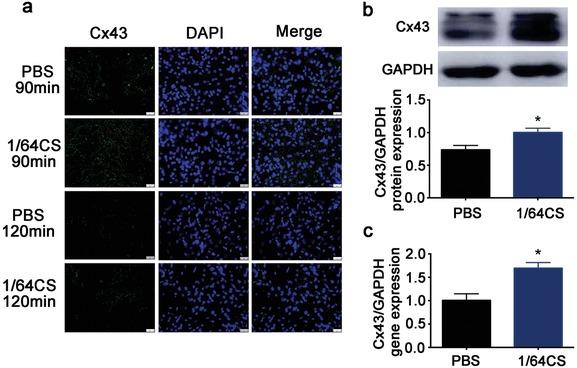
Effect of silicon‐enriched ion extract on the expression of gap junction associated Cx43 in NRCMs under glucose/oxygen deprived conditions in vitro. a) Representative images of Cx43 immunofluorescence staining of NRCMs cultured in silicon‐enriched ion extracts under glucose/oxygen deprived conditions for 90 and 120 min, respectively. Scale bars represent 25 µm. b) Protein expression of Cx43 measured by western blot and GAPDH was served as the loading control. c) Gene expression of Cx43 in NRCMs under glucose/oxygen deprived conditions measured by RT‐qPCR. All data are obtained from three independent experiments. *p < 0.01 versus PBS‐90 min; mean ± SD.

Gap junction associated Cx43 is known as an important protein in cardiomyocyte metabolism, and in particular it may play a protective role in myocardial infarction. Glucose/oxygen deprived resulted in a significant decrease of Cx43 expression of the NRCMs cultured for 120 min in PBS as compared to that of PBS‐90 min group. Silicon‐enriched ion extract significantly reduced the suppression effect of glucose/oxygen deprived on Cx43 expression in both 90 and 120 min groups as the fluorescence intensity of Cx43 in silicon‐enriched ion extract treatment group was remarkably enhanced (**Figure** **4**a). Moreover, both Cx43 protein and gene expression evaluated by Western blot and real‐time PCR analysis also confirmed the positive function of Si ions. We observed that silicon‐enriched ion extract significantly upregulated Cx43 protein and gene expression (Figure 4b,c). As Cx43 is widely expressed in heart gap junctions and is purported to play a crucial role in the synchronized contraction of the heart, these results indicate the potential roles of Si ions in promoting cell–cell communications thus enhancing cardiac function post‐AMI.

### 2.4 Effect of Silicon‐Enriched Ion Extract on Vascular Endothelial Growth Factor (VEGF)‐Mediated Angiogenesis of NRCMs and HUVECs Cocultures under Glucose/Oxygen Deprived Conditions In Vitro

**Figure 5:**
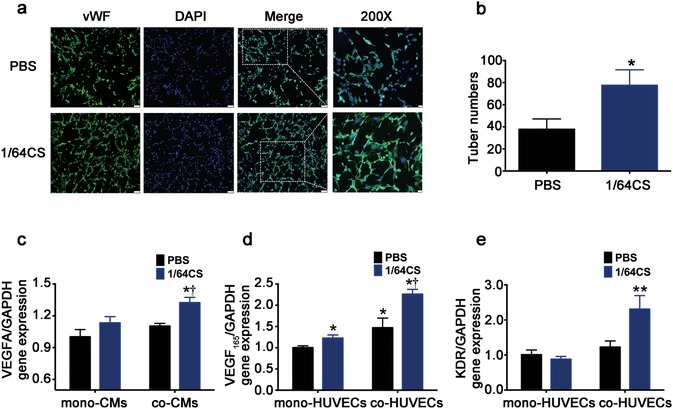
Effect of silicon‐enriched ion extract on VEGF‐mediated angiogenesis of NRCMs and HUVECs co‐cultures under glucose/oxygen deprived conditions in vitro. a) Representative fluorescence images of vWF‐stained tube formation of co‐HUVECs under glucose/oxygen deprived conditions in vitro. Scale bars represent 75 µm. b) Quantification of tune numbers in coculture systems (10 pictures for each group). *p < 0.01 versus PBS group; mean ± SD. c) Gene expression of VEGFA in mono and co‐NRCMs under glucose/oxygen deprived conditions. d) Gene expression of VEGF_165_ in mono and co‐HUVECs under glucose/oxygen deprived conditions. e) Gene expression of KDR in mono‐ and co‐HUVECs under glucose/oxygen deprived conditions for 120 min; *p < 0.05 versus PBS group. **p < 0.01 versus mono‐cultured PBS group; †*p* < 0.05 versus cocultured PBS. Data are obtained from three independent experiments; mean ± SD.

The proangiogenesis effect of silicon‐enriched ion extract was observed by using NRCMs and HUVECs cocultures under glucose/oxygen deprived environment in vitro. **Figure** **5**a,b shows that silicon‐enriched ion extracts of CS stimulated angiogenic responses in the coculture system. The vWF‐staining revealed that typical capillary‐like networks were formed in the cocultures both with and without bioactive ions, but it is much more evident in 1/64CS extract group as tube numbers formed in 1/64 CS extract group were markedly higher than that in control group. Next, in order to clarify whether the angiogenesis effect is mainly mediated by VEGF and whether this phenomenon was mainly functioned by paracrine effect, we explored the VEGF and kinase insert domain receptor (KDR) gene expression, the receptor of VEGF, in the monocultures and cocultures of NRCMs and HUVECs under glucose/oxygen deprived conditions. As shown in Figure 5c,d, in monocultured NRCMs no significant difference of VEGFA gene expression was seen between PBS and 1/64CS groups while in monocultured HUVECs, VEGF_165_ gene expression was increased in 1/64CS extract. In coculture system, VEGFA expression by co‐NRCMs and VEGF_165_ expression by co‐HUVECs were projected to increase in 1/64CS extract. Consist with VEGF expression results, KDR expression in co‐HUVECs was also increased correspondingly (Figure 5e). These findings also verify the confirmed view that paracrine effect plays a pivotal role in the recovery of myocardial post‐AMI.

### 2.5 Effect of “Ion Therapy” on Cardiac Function and Heart Remodeling as well as Hypertrophy Post‐AMI In Vivo

**Figure 6:**
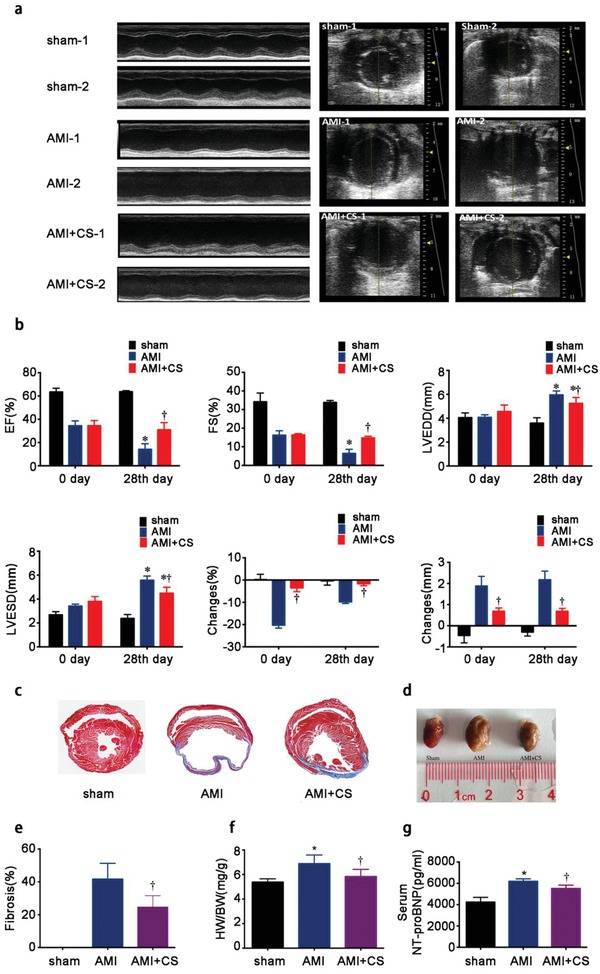
Effect of “ion therapy” on cardiac function and heart remodeling as well as hypertrophy post‐MI in vivo. a) Representative echocardiograms (left) and measurements of different groups obtained from the mid‐papillary muscle region of the left ventricle (right) of each groups before “ion therapy”(right after LAD ligation) and post “ion therapy” (4 weeks after LAD ligation); sham‐1,AMI‐1 and AMI+CS‐1: right after LAD ligation; sham‐2,AMI‐2 and AMI+CS‐2: 4 weeks after LAD ligation. b) Cardiac function measured by left ventricular end‐diastolic diameter (LVEDD), LV end‐systolic diameter (LVESD), the percentage of LV ejection fraction (EF) and fractional shortening (FS) values and changes before and post “ion therapy”. *p < 0.05 versus AMI group, †*p* < 0.01 versus AMI group. c) Representative Masson's trichrome staining of heart sections evaluating collagen deposition 4 weeks after surgery. d) Representative pictures of heart samples indicating hypertrophy degrees. e) Quantitative analysis of the area of fibrosis. Sham group was set as 0% and †*p* < 0.001 versus AMI group (10 pictures for each group). f) Heart weight/body weight (HW/BW) ratio in each groups. g) Serum expression of NT‐proBNP level in in each groups. Sham group *n* = 4; AMI group *n* = 7 and AMI+CS group *n* = 13. *p < 0.05 versus sham group, †*p* < 0.05 versus AMI group; mean ± SD.

In order to further verify our in vitro findings, investigating the protective effect of silicon ion on cardiomyocytes in vivo, and exploring the application possibility of the “ion therapy,” a mouse model of AMI was established and left ventricular (LV) function was evaluated by echocardiography right after the surgery and 4 weeks later. For grouping, the detailed information and method were described in the experimental section. In addition, there was also no significant difference in the level of myocardial enzyme spectrum between the AMI group and the AMI+CS group. The result of cardiac enzymatic expression was shown in Figure S5 (Supporting Information). The changes in echocardiography before and post “ion therapy” were investigated. To study the real therapeutic efficacy of “ion therapy,” different concentrations (1/2CS and CS extracts) of ion extracts were first test in a pre‐experiment. The results indicate that although both 1/2CS and CS ion extracts can improve cardiac function (Figure S6, Supporting Information) and alleviate fibrosis post‐AMI (Figure S7, Supporting Information), the CS group showed remarkably better therapeutic effect than 1/2CS group. Therefore, the CS extracts were chosen for further detailed in vivo experiments with increased animal numbers. As shown in **Figure** **6**a,b, right after LAD ligation, ejection fraction (EF) values of AMI mouse were decreased sharply to ≈40%. Compared to sham group, AMI mouse displayed remarkable LV enlargement indicating that the AMI mouse model were successfully established. After 28 d, EF values in AMI group were reduced by 20% when compared with that right after LAD ligation. However, the EF values in CS group did not show significant change after same time period. The similar results were also observed in fractional shortening (FS) values. Additionally, EF and FS values in CS group were significantly higher than those of AMI group after injection. These results indicate that cardiac function deteriorates slowly after myocardial infarction and CS can alleviate this decrease. Indeed the results revealed that the “ion therapy” significantly improved cardiac systolic and diastolic functions. Compared to AMI group, intravenous injection obviously alleviate the deterioration of contractile function of hearts post‐AMI defined by the changes of FS, EF, left ventricular end‐diastolic diameter (LVEDD), and LV end‐systolic diameter (LVESD) values. These results were also verified by histological Masson's trichrome staining of collagen deposition. Compared to AMI group, the “ion therapy” showed remarkably decreased content of collagen (Figure 6c,e). In accordance, heart samples size, serum NT‐proBNP level, heart to body weight ratios “ion therapy” group were also significantly decreased than that of AMI group (Figure 6d,f,g). These data indicate that the “ion therapy” clearly have therapeutic effect on AMI mice by attenuating left ventricular remodeling and hypertrophy.

### 2.6 Effect of “Ion Therapy” on Cardiac Cell Apoptosis In Vivo Post‐AMI

**Figure 7:**
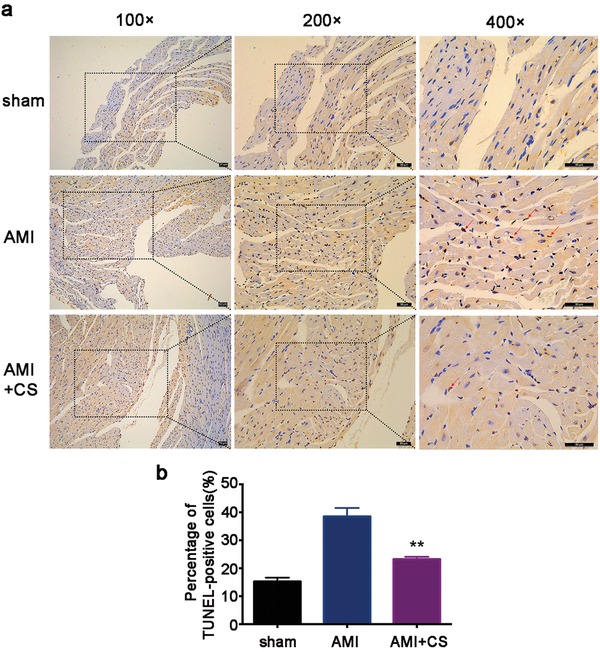
Effect of “ion therapy” on cardiac apoptosis in vivo post‐AMI. a) Representative immunohistochemical pictures of TUNEL staining in border‐zone of infarction. Total nuclei (DAPI staining, blue) and TUNEL positive nuclei (brown‐yellow). Red arrows show TUNEL‐positive cardiomyocytes. Scale bar represents 50 µm. b) Quantitative analysis of TUNEL‐positive cardiomyocytes (10 pictures for each group). Sham group *n* = 4; AMI group *n* = 7 and AMI+CS group *n* = 13. **p < 0.01 versus AMI group; mean ± SD.

Considering the in vitro antiapoptosis effect of Si ions, we also investigated the antiapoptosis effect of “ion therapy” on AMI by using TUNEL assay on histological sections of AMI animals in vivo. As shown in **Figure** **7**a, the number of TUNEL‐positive nuclei (stained in brown‐yellow ) in AMI mice was clearly more than that of sham group suggesting that ischemic condition induced apoptosis of cardiomyocytes in heart tissue. In contrast, the CS ions treated group showed a clear decrease of the TUNEL‐positive staining. The quantitative analysis of the assay further confirmed the observation that the percentages of TUNEL‐positive stained cells in CS extracts treatment groups, namely the “ion therapy” group, declined sharply as compared to that of AMI group (Figure 7b). Considering our in vitro findings, it is clear that the silicon ion extract of the “ion therapy” has the function to protect cardiac cells and reduce myocardial apoptosis in AMI.

### 2.7 Effect of “Ion Therapy” on the Expression of Gap Junction Associated Cx43 in Cardiomyocytes In Vivo Post AMI

**Figure 8:**
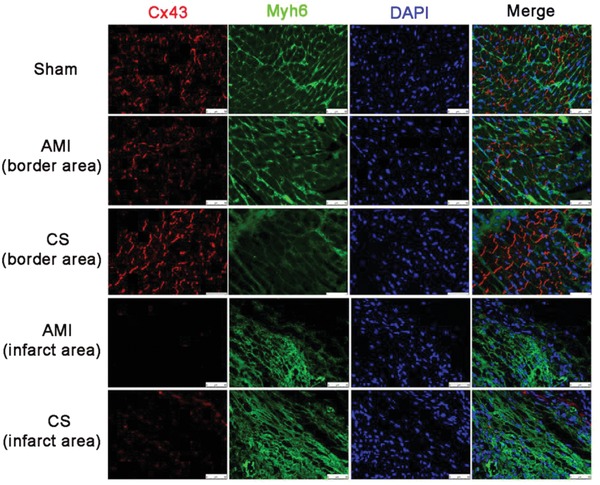
Effect of “ion therapy” on the expression of gap junction associated Cx43 in cardiomyocytes in vivo post‐AMI. Representative immunofluorescence images of Myh6 (green) and gap junction associated Cx43 (red) staining in the infarcted myocardium 4 weeks after surgery. Scale bars represent 50 µm.

To study the effect of “ion therapy” on promoting cell–cell communications in AMI mice hearts, protein level of Cx43 were investigated. Myh6 protein is expressed specifically and predominantly in cardiomyocytes and is the major protein comprising the cardiac muscle thick filament, and functions in cardiac muscle contraction. In this study, Myh6 staining was used to distinguish cardiomyocytes to other cells. It is clear to see that myh6 protein structure and arrangement in AMI mice were disorganized and some even disappeared both in the infarcted area and border area as compared to sham group (Figure S3, Supporting Information). As expected, Cx43 proteins were abundantly expressed in the intercalated disc (Figure S3, Supporting Information and **Figure** **8** merge pictures). In normal heart (sham group), Cx43 was widely expressed while the expression was downregulated in AMI mice (both in infarcted area and border area). In the border area, Cx43 was slightly decreased while in infarct area the decrease was much more evident suggesting that ischemic condition indeed has great influence on Cx43 expression in AMI. Interestingly, the immunofluorescence staining showed that the “ion therapy” treatment (CS group) clearly increased Cx43 expression both in infarct area and border area as compared to that of AMI group (Figure 8). The in vivo results confirmed our in vitro findings and indicates that the “ion therapy” has the activity to promote cell–cell communications post AMI.

### 2.8 Effect of “Ion Therapy” on VEGF‐Mediated Angiogenesis in Border Area of Ischemic Tissues In Vivo Post AMI

**Figure 9:**
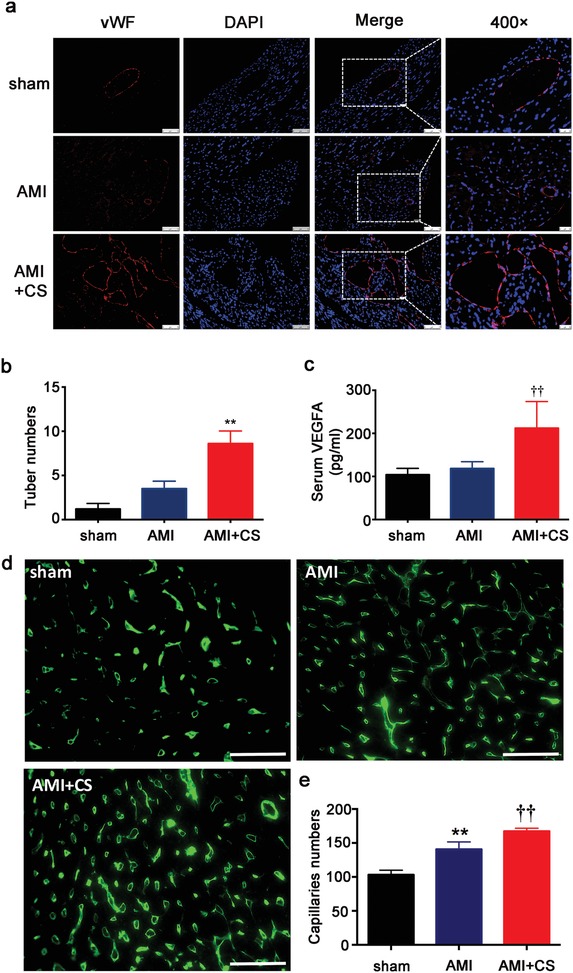
Effect of “ion therapy” on VEGF‐mediated angiogenesis in border area of ischemic in vivo post‐ AMI. a) Representative immunofluorescence images of vWF‐stained blood vessels in the border area of infarcted myocardium 4 weeks after surgery. Scale bars represent 75 or 50 µm, respectively. b) Quantification of tube number/HPF in the border regions of infarctions (10 pictures for each group). **p < 0.01 versus AMI group; mean ± SD. c) Serum VEGFA expression post‐AMI. d) Representative immunofluorescence images of isolectin IB4‐stained capillaries in the border area of infarcted myocardium 4 weeks after surgery. Scale bars represent 50 µm. e) Quantification of capillaries numbers/HPF in the border regions of infarctions (five pictures for each group). **p < 0.01 versus sham group, ††*p* < 0.01 versus AMI group. Sham group *n* = 4; AMI group *n* = 7 and AMI+CS group *n* = 13; mean ± SD.

One of the most critical issue in AMI treatment is to rebuild blood supply, which was damaged under ischemic condition. Therefore, the in vivo effect of the “ion therapy” on angiogenesis was investigated by using vWF (large blood vessels) and isolectin‐IB4 (for capillaries) immunofluorescence staining of heart tissues after different treatments. **Figure** **9**a,d illustrates that “ion therapy” stimulated angiogenic responses in the border area of infarction. More blood vessels and capillaries were observed in the “ion therapy” group as compared to AMI group. Quantitative analysis further confirmed that the blood vessel and capillary numbers of “ion therapy” group were significantly increased as compared to AMI group (Figure 9b,e). To further confirm the angiogenic effect of bioactive ions, we analyzed serum VEGFA level by using a ELISA assay. The results showed that the serum level of VEGFA in AMI group was slightly higher than that of sham group due to ischemic stimulation of the AMI condition, which induced VEGF‐mediated neovascularization in the border area of infarction myocardial (Figure 9c). Interestingly, it is observed that serum VEGFA level in “ion therapy” group was significantly increased as compared to AMI group (Figure 9c). Taken together, our in vivo findings demonstrated that the “ion therapy” indeed activated angiogenesis and promoted blood vessel formation in AMI animals.

### 2.9 Biodistribution and Metabolism of Si and Acute Toxicity of “Ion Therapy”

**Figure 10:**
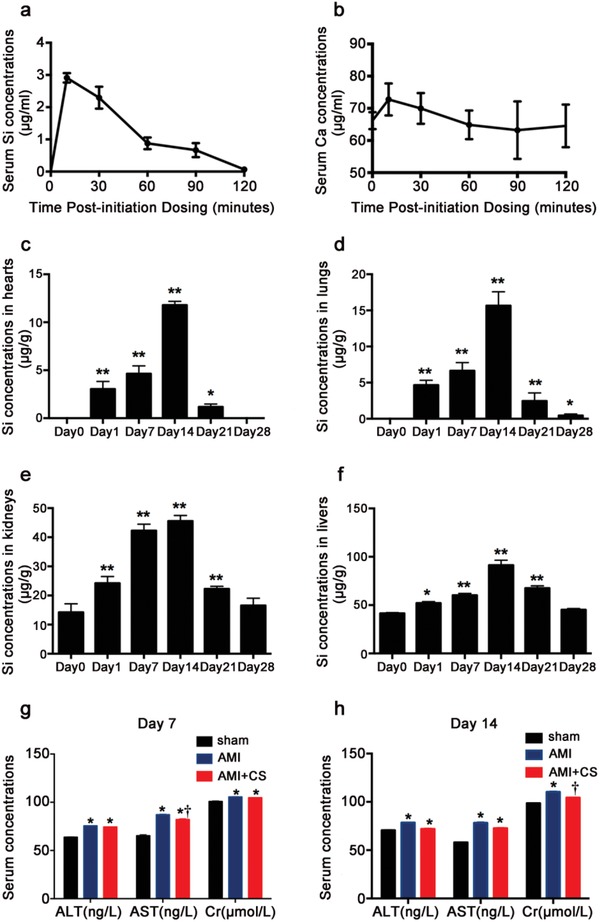
Bio‐distribution and metabolism of Si and acute toxicity of “ion therapy.” a) Serum Si concentrations after initiation injection in AMI mouse (*n* = 4). b) Serum Ca ion concentrations after initiation injection in AMI mouse (*n* = 4). c) Si concentrations in hearts before and after “ion therapy” for 1, 7, 14, 21, and 28 d (*n* = 3 for each group). d) Si concentrations in lungs before and after “ion therapy” for 1, 7, 14, 21, and 28 d (*n* = 3 for each group). e) Si concentrations in kidneys before and after “ion therapy” for 1, 7, 14, 21, and 28 d (*n* = 3 for each group). f) Si concentrations in liver before and after “ion therapy” for 1, 7, 14, 21, and 28 d (*n* = 3 for each group). g) Serum expression of ALT, AST, and Cr level in each groups after injection for 7 d. h) Serum expression of ALT, AST, and Cr level in each groups after injection for 14 d. Sham group *n* = 4; AMI group *n* = 7 and AMI+CS group *n* = 13. *p < 0.05 versus sham group or day 0, **p < 0.01 versus Day 0, †*p* < 0.05 versus AMI group; mean ± SD.

Si concentrations in blood and important organs including heart, lung, liver and kidney before and postinjection were measured, and the results are shown in **Figure** **10**. The metabolism time of Si in blood was fast. The Si concentration was less than 1 ppm in serum before injection (at 0 min), increased after ion injection, reached maximum after 10 min, and then decreased quickly with time. After 120 min, the Si ion concentration was almost back to the level before the injection (Figure 10a,b). In all organs such as heart, liver, lung, and kidney, the Si ion concentrations changed in a similar pattern with time after injection, which increased after injection and reached maximum at day 14, and then decreased back to the normal level at day 28, indicating no significant accumulation of Si in all important organs (Figure 10c–f). It is worth to indicate that after injection Si ion concentration increased in heart indicating that Si ions indeed were infiltrated in heart tissue through intravenous injection. Then, to investigate whether these increased trace element content in organs would harm the functions of the organs, the acute toxicity of “ion therapy” was investigated. As shown in Figure 10g,h, the results revealed that no acute inflammation reaction was observed as no significant differences in serum aspartate aminotransferase (AST), alanine aminotransferase (ALT), and creatine kinase (CK) level between AMI and AMI+CS groups at day 7 and day 14.

## 3 Discussion

**Figure 11:**
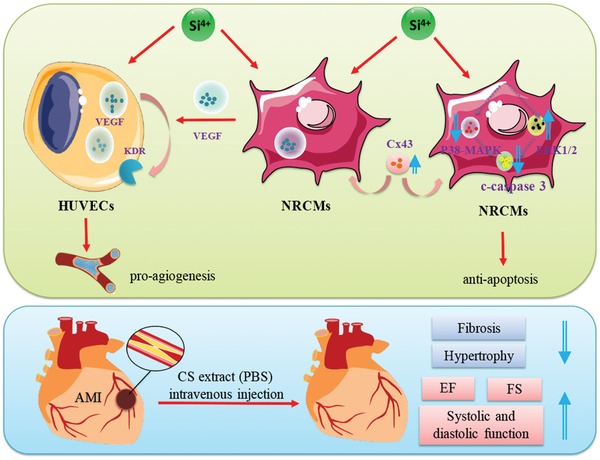
Overall effects and mechanism of “ion therapy” on AMI treatment. “Ion therapy” can significantly improve cardiac function in mice post‐AMI by stimulating Cx43 mediated gap junction thus promoting VEGF‐mediated angiogenesis and by inhibiting caspase 3‐associated apoptosis.

AMI is the acute necrosis of myocardial tissue, which threats the public health seriously.^16^ Previous tissue engineering therapies for AMI treatment has acquired certain effect but^17^, ^18^, ^19^, ^20^, ^21^, ^22^ few observed robust myocardial regeneration and focused on the paracrine effects of angiogenesis and antiapoptosis effects stimulated by stem cells.^20^ Here in this study, we proposed, for the first time, a new treatment concept for AMI therapy utilizing bioactive ions, which are bioactive components of bioceramics. By intravenous injection, the bioactive ions from biomaterials were delivered into the infarct area, which not only makes up for the deficiency of traditional medicine for AMI treatment in endogenous recovery, but also avoids drawbacks of in situ cell injection or local surgical cell/biomaterial implantation. Our results demonstrated that the “ion therapy” can improve cardiac function by promoting cell–cell communication, enhance gap junction associated Cx43 expression thus stimulate VEGF mediated angiogenesis, reconstruct blood flow and inhibit the MAPK family protein‐associated apoptosis (**Figure** **11**). Furthermore, it is for the first time revealed that bioactive ions such as Si are able to regulate cardiomyocyte behavior and stimulate angiogenesis during AMI recovery. This “ion therapy” not only provides a novel strategy for the treatment of AMI, but also give new insight for the application of biomaterials in systemic treatment of human diseases.

Cardiac myocytes apoptosis is a key factor in the pathogenesis of myocardial infarction and acute coronary artery occlusion is often accompanied by myocardial apoptosis. Inhibition of apoptosis has been considered as an effective treatment for AMI as hypoxia induced cardiomyocyte apoptosis plays an important role in the development of heart failure post‐AMI.^23^, ^24^ This study shows that bioactive silicon ions not only promoted angiogenesis, but also played an active role in inhibiting cardiomyocyte apoptosis and enhancing myocardial viability. In vitro, the results of cell viability and gene expression indicated that silicon ions enhanced cardiomyocyte viability and stimulated expression of cardiomyocyte specific marker genes under normal and glucose/oxygen deprived conditions. In addition, the results of TUNEL staining and c‐caspase 3 protein expression indicated that Si ions may play a protective role on NRCMs by inhibiting apoptosis. To further explore the underlying mechanisms, we investigated the expression of apoptosis‐related MAPK family proteins in NRCMs under glucose/oxygen deprived conditions. Our findings showed that the expression of MAPK p38 and ERK 1/2 was regulated by Si ions, in which the phosphorylated p38 was downregulated while phosphorylated ERK1/2 was upregulated. Previous studies have found that selective inhibition of MAPK p38 or activation of MAPK ERK 1/2 pathway can reduce apoptosis induced by ischemic injury. Our previous studies have also revealed that silicate‐based biomaterials activated the AMPK/ERK1/2 and PI3K/AKT signaling pathways^25^ in the process of osteogenesis. The extract of β‐CS/PDLGA significantly increased the expression of p‐ERK 1/2 in rBMSCs and the expression of p‐AKT in HUVECs. In this study, we demonstrated that the bioactive Si ions simultaneously inhibited expression of phosphorylated p38 and activated expression of ERK1/2 of NCRMs, which suggests a highly effective regulation of Si ions on NCRMs apoptosis. However, in this study, no significant difference was seen in the expression of phosphorylated AKT in silicon ions treated group. This may because of different treatment conditions and cell types. In previous study, the expression of p‐AKT in HUVECs was investigated under normal or hypoxia condition whereas in this study the expression of p‐AKT in NRCMs under glucose/hypoxia condition were estimated. Overall, it can be speculated that silicon ions may play a role in antiapoptosis by affecting the expression of MAPK proteins in NRCMs under glucose/oxygen deprivation conditions. By comparing ion concentrations between the culture medium and the control medium (PBS), it is confirmed that Si ions (the most effective concentration is 4.53–9.08 µg mL^−1^) play a major role in the antiapoptotic effect for NRCMs under glucose/oxygen deprived conditions. Most importantly, the in vivo results demonstrated that the “ion therapy” treatment lead to significantly decreased TUMEL‐positive cell staining in infarct tissue, and confirmed the inhibitory effect of bioactive Si ions on NRCMs apoptosis in vivo. However, the in vivo model could not distinguish whether the protection effect of “ion therapy” on cardiomyocyte survival results from the stimulation of angiogenesis or the direct prosurvival effect of the ions or both. It is important to clarify this issue in the future research. Additionally, in this study we only focused on the effect of Si and Ca ions, so we selected calcium silicate, one of the simplest silicates, which only contains Si, Ca, and O elements. However, previous reports have shown that some other elements such as potassium,^26^ zinc,^27^ lithium,^28^ and magnesium^29^ may affect the function of heart. Possible synergistic effect of these trace elements with Si ions in “ion therapy” should be considered in further studies.

Recovery of blood perfusion can rescue the dying cardiomyocytes, reduce ventricular remodeling infarction, protect cardiac structure, reduce myocardial fibrosis and cardiomyocyte hypertrophy, and improve cardiac function. Re‐establishment of blood flow in infarct tissue is similar as irrigating the arid farmland, which can save the dying cardiac cells affected by AMI condition.^30^ The formation of neovascularization is essential for revascularization post‐AMI. The evidence that the bioactive ions released from silicate biomaterials can promote endothelial cells to form blood vessels has been clearly identified in our previous studies.^11^, ^12^, ^31^ Li et al.^13^ demonstrated that the CS extract (mainly containing Si and Ca ions) promoted the interaction between fibroblasts and endothelial cells through paracrine effect and stimulated the formation of blood vessels. However, whether bioactive silicon ions can regulate intercellular communication between cardiomyocytes and further affect endothelia cells behavior under glucose/oxygen deprived conditions and promote angiogenesis remain unclear. In order to test our hypothesis that the bioactive Si ions may enhance angiogenesis in infarct tissue through activating cell–cell communication, we first investigate the expression of gap junction related Cx43 in cultured NRCMs under glucose/oxygen deprived conditions. Cx43 is the most abundant and widely expressed connexin family protein in atrial and ventricular myocytes.^32^ It has been reported that mitogen activated protein kinases (MAPK) such as p42/p44 and p38 might be involved in the regulation of Cx43 expression and phosphorylation.^33^, ^34^, ^35^ Increased Cx43 phosphorylation is mediated by p38 in neonatal rat ventricular cardiomyocytes.^34^ In this study, we found that Si ion upregulated Cx43 expression in NRCMs with the increased phosphorylation level of p38 and p44/p42. Therefore, it is with high possibility that Si ion may affect gap junction and upregulate Cx43 expression by regulating the phosphorylation level of p38 and p44/p42. This is in consistence with our previous finding that the CS extract exerts protective effects via stimulating the expression of Cx43 between endothelial cells under hypoxia conditions,^10^ but further study is needed to elucidate the detailed mechanisms of Si ion activation of NRCMs. Next, we tried to clarify whether silicon ions can regulate endothelial cells behavior and promote VEGF mediated angiogenesis via paracrine effect by affecting closely connected cardiomyocyte under glucose/oxygen deprived conditions. We investigated the expression of VEGF in mono‐ and cocultures of NRCMs and HUVECs system under glucose/oxygen deprived conditions. Interestingly, we found that Si ion can increase the expression of VEG_165_ in mono‐HUVECs while no significant change of VEGF expression in mono‐NRCMs was observed. However, in cocultured NRCMs, Si ions significantly stimulated the VEGF expression. In addition, higher expression of KDR in co‐HUVECs was also observed, suggesting that the co‐NRCMs might activate the expression of KDR in cocultured HUVECs via paracrine effect. In our previous study, we have identified similar paracrine activation of Cx43 expression in hypoxia HUVECs and enhancement of the communication between endothelial cells by Si ions released from dressing materials in wound healing.^10^ Being the important “logistics support” for working heart, endothelial cells are critical for angiogenesis and blood vessel formation. The vitality of the endothelial cells directly affects the function of the blood vessels thus influences the vitality of working cardiomyocytes. Here in this study, the similar paracrine effect has also been observed between cardiomyocytes and endothelial cells for angiogenesis. In myocardial infarction, the protection of cardiac myocytes and endothelial cells (in vitro that is glucose/oxygen deprived conditions) is essential and crucial for recovery of cardiac tissues from AMI condition. Here we demonstrated that the bioactive Si ions also activated interactions between cardiomyocytes and endothelia cells, and promote angiogenesis. And most importantly, not only in vitro, but also in vivo, we found that intravenously injected silicon ions were functioning as a “protector” and “activator” for both cardiomyocytes and endothelial cells in the damaged heart by promoting cell‐cell communications, and finally resulted in the re‐establishment of blood supply in AMI.

In the past decade, silicate materials have been developed as implants for bone regeneration and wound healing.^10^, ^36^, ^37^ However, it is unknown whether the bioactive function of these local applied biomaterials can be utilized for systemic application such as AMI treatment through intravenous injection of material derived bioactive ions. The fundamental scientific question is whether active silicon ions can be transported to AMI site through venous blood vessels and play bioactive roles in protecting cardiomyocytes and promoting angiogenesis. This investigation confirmed our hypothesis that the intravenous injection based “ion therapy” does have a significant effect on myocardial infarction. Using a mouse model of myocardial infarction, our results demonstrated that silicon ions derived from calcium silicate bioceramics indeed can function in myocardial infarction area by intravenous injection, promote neovascularization, restore blood flow, reduce the degree of myocardial injury, prevent heart failure, and thus significantly improve cardiac function. The therapeutic effect are clearly supported by the changes of myocardial systolic and diastolic function (EF, FS, LVEDD, and LVEDS), and the results of histology, enzymology and morphology. The findings of Masson staining showed that silicon ion can significantly reduce the myocardial fibrosis, inhibit scar formation and reduce fibrosis. Immunohistochemical staining demonstrated that silicon ions enhanced angiogenesis in vivo, provided nutrition and oxygen for damaged heart tissues and promoted endogenous recovery. Additionally, by immunofluorescence staining for specific marker of myocardial cells in vivo, we revealed that “ion therapy” enhanced cell–cell interaction in AMI area as Cx43 expression remarkably increased in Si ion treated group. These results demonstrated that intravenous injected bioactive silicon ions was able to penetrate into the infarcted site and affect cells in the border area of infarcted site, confirming that “ion therapy” indeed has therapeutic effects.

Silicon, as an important micronutrient trace element in human and animal tissues, is a constituent of some glycosaminoglycans and polyuronides, which can bind to the polysaccharide matrix tightly.^38^ It is mainly distributed in connective tissue, bones, tendons, muscles, hair, feathers, and skin.^39^, ^40^ Lacking of silicon may lead to abnormal bone and cartilage formation as well as other diseases. Based on our previous findings on activity of Si ions in stimulating cell viability and angiogenesis, we propose “ion therapy” as a novel strategy for AMI. One of the key issues is whether these bioactive ions can be delivered to heart tissue through intravenous injection. By the measurement of ion concentrations in organs, we found that Si concentration in heart increased over time which indicating that the injected Si ions were indeed delivered to the heart tissue indicating that “ion therapy” is achievable by intravenous injection. Another important issue is the safety of the ion injection. It is known that, as compared to other medication ways such as enteral and topical administration, intravenous injection can avoid the first pass elimination effect and increase the bioavailability with fast metabolism time. Our results on serum ion concentration analysis revealed that the metabolism time of Si was very fast by intravenous injection. No Si in blood was detected before injection, which was in consistent with previous reports showing that the Si concentrations is less than 1 ppm in serum.^39^, ^41^, ^42^ After injection, although the Si concentration in blood increased sharply first, but then falls back to normal level in 120 min and the peak concentration was 3 ppm, at which no acute toxicity was observed. Analysis of Si concentrations in other important organs such as heart, lung, kidney, and liver was also similar as the result in blood. After injection, Si concentrations in organs first increased with time, reached a maximal concentration at day 14, and fell back to normal level in 3–4 weeks. This result indicates no Si accumulation in the major organs, and the possibility of induced long‐term toxicity and side effects is low. Furthermore, our evaluation of the acute toxicity of “ion therapy” revealed no acute inflammation reaction 7 and 14 d after injection, and no significant differences of Si concentrations in heart, liver, lung, and kidney were found as compared to control group without injection at day 28 indicating that the “ion therapy” is safe. These results in some degree are consisted with our previous findings on Si concentration in kidney, liver, lung, and spleen after CS scaffold implantation in bone defect for 4, 8, and 12 weeks, and Si ions in the body are nontoxic, are maintained within physiologically safe range and the excess Si was finally excreted through the urine.^39^, ^41^ Considering all of these results, we demonstrated that “ion therapy” is an effective and safety strategy for cardiac tissue engineering. However, although we have investigated the acute toxicity by estimating liver enzymatic expression and kidney creatinine level, the long‐term toxicity has not been investigated, in particular the long‐term organ function such as renal function after follow‐up, which should also be considered in the future.

## 4 Experimental Section

*Synthesis of CS Powders*: CS powder was prepared by using a chemical coprecipitation method as previously described.^43^, ^44^ At room temperature, by continuously mixing aqueous solution of Na_2_SiO_3_ (1 mol L^−1^) and Ca (NO3) _2_ (1 mol L^−1^) for 24 h, the reaction mixture with a molar mass of Na_2_SiO_3_:Ca (NO_3_) _2_ = 1:1 was obtained. The mixture was then filtered and washed thoroughly with deionized water and ethanol to give a CS suspension. Then, after drying at 80 °C overnight and calcining at 800 °C for 2 h, the resulting CS powder was sieved to obtain 100–150 µm particles for future use.

*Ion Extract Preparation and Ion Concentration Determination*: According to previously reported procedures,^44^, ^45^, ^46^ the ion extracts of CS bioceramics were prepared by soaking 1 g of CS powder in 5 mL of PBS or serum‐free DMEM (GIBICO) and incubated at 37 °C for 24 h. The suspension of CS powder was then centrifuged to collect the supernatant. A filter (Millipore, 0.22 µm) was used to sterilize the supernatant and serial dilutions of extracts (1/2, 1/4, 1/8, 1/16,1/32, 1/64, 1/128, and 1/256) were prepared by using PBS or cardiomyocyte growth medium [DMEM+10% fetal bovine serum(FBS)+1% P/S (penicillin/streptomycin)] respectively and stored at 4 °C for future use. ICP‐AES was used to determine the concentrations of the Ca, P, and Si ions in the solution.

*Cells Isolation and Culture*: Neonatal Sprague‐Dawley (SD) rats (1–3 d old, weighing 5–7 g, means 6.1 ± 0.7 g) were obtained from the Experimental Animal Centre of Southern Medical University. Primary neonatal rat cardiomyocytes (NRCMs) were isolated from hearts of neonatal SD rats as previously reported^47^ and the purity of the isolated cardiomyocytes was determined by immunofluorescence staining of cTnT (cardiomyocytes specific marker,Abcam,ab6994) and vimentin(fibroblasts specific marker, Abcam,ab11370). The isolated NRCMs with purity as high as 93% were used for further studies (Figure S1, Supporting Information). After separation from fibroblasts, enriched cardiomyocytes were seeded at density of 1.5 × 10^4^ cell per well in 96‐well plates or 5 × 10^5^ cell per well in six‐well plates in cardiomyocyte growth medium for 96 h before use. HUVECs were isolated according to previously described methods^48^ and the obtained HUVECs were cultured in endothelial cell medium (ECM) for 12 h before use. HUVECs at passages from 3 to 5 were used in this study. Cells were seeded at a density of 40 000 cells cm^−2^ in six‐well plate for future experiments. Cells at ≈80% confluence were prepared for future experiments. To investigate the effect of Si ions on the proliferation of cells, NRCMs were cultured under normal condition for 5 d and under glucose/hypoxia deprivation condition for 120 min, respectively. The expression of NRCMs specific marker genes such as were investigated.

For NRCMs and HUVECs coculture experiments, the transwell permeable supports (Corning, 12 mm Diameter Insert, 12 Well) with a 0.4 µm polycarbonate membrane were used in the coculture system to separate HUVECs and NRCMs into different compartments. NRCMs were plated at a density of 5 × 10^4^ cell per well in top chamber of a transwell insert and monocultured in cardiomyocyte growth medium for 96 h before use. HUVECs were seeded at a density of 40 000 cells cm^−2^ in the lower chamber and monocultured in ECM for 12 h before use. After that, NRCMs were precultured in top chamber of a transwell insert and HUVECs precultured in the lower chamber were combined and further cocultured under hypoxia conditions (94% N_2_, 5% CO_2_, and 1% O_2_) with PBS or CS extracts diluted in PBS in an anaerobic system (Thermo Forma, Marietta, OH, USA) at 37 °C for 90 or 120 min.

*Cell Viability Detection*: To determine the effect of CS extracts on cardiomyocytes viability in vitro under normoxia and glucose/oxygen deprived conditions, serial dilutions of CS extracts (1/2, 1/4, 1/8, 1/16,1/32, 1/64, 1/128, and 1/256) diluted in cardiomyocyte growth medium or PBS were prepared. At day 1, day 3, and day 5, the effect of CS extracts (diluted in NRCM growth medium) on NRCMs cell viability was detected under normoxia condition. For glucose/oxygen deprivation, NRCMs were cultured in CS extracts (diluted in PBS) under hypoxia condition for 90 or 120 min and cell viability were detected. The NRCM viability was measured by CCK8 assays. The medium was removed and replaced with 110 µL of DMEM medium or PBS containing 10 µL of CCK8 solution (Cell counting kit‐8, Dojindo, Kumamoto, Japan) according to the manufacturer's instructions. The absorbance was measured spectrophotometrically using a microplate reader (Bio‐Rad Benchmark Plus) at wavelengths of 450 nm.

*Determination of NRCMs Apoptosis In Vitro*: For glucose/oxygen deprivation, NRCMs were cultured in PBS or 1/64 CS extract (diluted in PBS) under hypoxia conditions for 90 or 120 min. The cells were then fixed in 4% paraformaldehyde and apoptotic cells were detected by TUNEL staining using one‐step TUNEL Apoptosis Assay Kit (Beyotime, Jiangsu, China) according to the manufacturer's protocol. Cells were counterstained with DAPI (Sigma–Aldrich, St. Louis, MO, USA) and TUNEL positive cells were observed by a fluorescence microscope (Leica DMI8, Germany). Total nuclei (DAPI staining, blue) and TUNEL positive nuclei (green) in each field were counted in ten randomly chosen field, and the index of apoptosis (number of TUNEL‐positive nuclei/total number of nuclei × 100%) was calculated.

*DCFH‐DA Staining for Analysis of Intracellular ROS Activity Level*: NRCMs (1 × 10^4^ per well) were seeded in black bottomed 96‐well culture plate in PBS or 1/64 CS extract (diluted in PBS) under hypoxia conditions for 120 min. After treatment, cells were incubated with 10 × 10^−3^ m DCFH‐DA for 30 min at 37 °C. After washing with PBS for three times, fluorescence intensity was measured with a multiwell microplate reader at an emission wavelength of 528 nm and at an excitation wavelength of 488 nm. All the values were expressed as percentage fluorescence intensity relative to the control.

*Protein Isolation and Western Blot Analysis*: In vitro, the protein level of Cx43, AKT, *p*‐AKT, MAPK p38, MAPK *p*‐p38, Erk1/2, *p*‐Erk1/2, and *p*‐JNK were determined by Western blot analysis. Following treatment, cells were washed with cold PBS (pH 7.4) and lysed for 15 min with RIPA lysis buffer (Beyotime, Nantong, China) supplemented with a cocktail of protease and phosphatase inhibitors (Sigma Chemical Co, St Louis, MO) in ice bath and isolated from the following standard protocol. Protein concentrations were determined by the bicinchoninic acid (BCA) method. Each sample (50 µg of protein per lane) was separated by sodium dodecyl sulfate–polyacrylamide gel (10%) electrophoresis followed by electrophoretic transfer of protein from the gel to a nitrocellulose membrane. The membranes were then treated with the blocking buffer for 1 h at room temperature, followed by incubation with primary antibodies at 4 °C overnight. Primary antibodies used here included anti‐Cx43(Abcam,ab11370), anti‐p44/42 MAPK(Erk1/2) (1:1000 dilution, CST,4695P), anti‐Phospho‐p44/42 MAPK (Thr202/Tyr204) (1:1000 dilution, CST,9101S), anti‐Phospho‐p38 MAPK (Thr180/Tyr182) (1:1000 dilution, CST, 4511S), anti‐p38 MAPK(1:1000 dilution, CST,8690S), anti‐Phospho‐AKT (Ser473) (1:1000 dilution, CST, 4060), anti‐AKT (1:1000 dilution, CST, 9272), anti‐Phospho‐SAPK/JNK (Thr183/Tyr185)(1:1000 dilution,CST,4668), and anti‐cleaved caspase‐3(1:1000 dilution, CST, 14220S). Normalization of results was conducted by running parallel Western blots for detecting glyceraldehyde 3‐phosphate dehydrogenase protein (GAPDH). The optical density was quantified using an image processing analysis program.

*Quantitative Real‐Time Polymerase Chain Reaction* (Q‐RT‐PCR): For RNA extraction, cells were prepared as described before. Total RNA was extracted from cultured cells or total left ventricular tissue using the TRIzol (Invitrogen) reagent and following the manufacturer's protocol. The concentration of RNA was measured though a nanodrop 1000 reader (Thermo Scientific). cDNA was synthesized using a ReverTra Ace‐a kit (Takara, Japan) according to the manufacturer's instructions. Primer (all from Sangon Biotech Co. Ltd.) were used as the final concentration of 400 × 10^−9^ m. Glyceraldehyde 3‐phosphate dehydrogenase (GAPDH) was used as a housekeeping gene. The sequences are shown in Table S1 (Supporting Information). Reactions were performed in triplicate. Data were analyzed with the SDS 2.3 software and compared by the ΔΔC_t_ method, and each Q‐RT‐PCR was performed in triplicate for yield validation. Data were normalized to GAPDH mRNA expression of each condition and were quantified relative to the corresponding gene expression from control sample (cells cultured with PBS) at 90 or 120 min, which were standardized to 1.

In vitro vWF,Cx43,cTnT and vimentin immunofluorescence staining. In vitro, immunofluorescence staining of von Willebrand factor (vWF) was applied on HUVECs and NRCMs cocultures under glucose/oxygen deprivation conditions. Meanwhile, Cx 43 immunofluorescence staining was also applied in NRCM monoculture under glucose/oxygen deprived conditions. The cells were incubated either with PBS or PBS with 1/64 CS extracts under hypoxia conditions in an anaerobic system (Thermo Forma, Marietta, OH, USA) at 37 °C for 90 or 120 min. After that, the co‐HUVECs or mono‐NRCMs were fixed for 15 min in 4% paraformaldehyde at room temperature and permeabilized with 0.5% Triton X‐100 (PBS) for 30 min and blocked with PBS containing 1% bovine serum albumin (BSA) for 1 h at 37 °C. The immunofluorescence staining was carried out using rabbit anti‐vWF (Abcam,ab6994) or rabbit anti‐connexin 43(Abcam,ab11370) diluted in 0.5% BSA at 1/500 according to the manufacturer's instruction. To reveal the vWF or Cx43, cells were incubated with Alexa Fluor 488 goat anti‐rabbit IgG (Santa Cruz) or cy3 goat anti‐rabbit IgG (Santa Cruz), and then counterstained with DAPI (Sigma–Aldrich, St. Louis, MO, USA) and pictures were taken by using a fluorescence microscope (Leica DMI8, Germany). Ten randomly chosen images were taken per well, and tubules were manually counted and averaged over the ten images.

*Mouse AMI Modeling and Grouping*: Adult male C57 BL/6 mice (8–10 week old) were purchased from laboratory animal center of Southern Medical University. Protocols were approved by the Southern Medical University animal care and use committee guidelines, which conform to the guide for the care and use of laboratory animals published by the US National Institutes of Health (8th edition, 2011). For the AMI model, mice were subjected to permanent left anterior descending (LAD) ligation as described previously.^49^ In brief, the animals were anesthetized using tribromoethanol (1.2%, 0.02 mL g^−1^) and ventilated using a rodent ventilator (DW‐3000B; Xinsida, Beijing, China) with 100 breaths per min and a stroke volume of 100 mL. A left thoracotomy was performed, and the LAD was ligated using an 8‐0‐prolene suture with a mortality rate of 80%. Sham group mice underwent the same surgical procedure with the exception that the LAD was not ligated. Echocardiography was performed right after LAD ligation and the animals were mainly randomized based on echocardiography. The mouse with LAD ligation treatment were randomly divided into AMI (*n* = 7) and AMI+CS (*n* = 13) group by matching the values of cardiac function index. There were no differences in EF, FS, LVEDD, and LVESD values between AMI and AMI+CS groups before “ion therapy.” In addition, no differences were seen in serum cTnT and CK‐MB level between AMI and CS groups. The results of cardiac enzymatic level were shown in Figure S5 (Supporting Information). After that, the AMI+CS group received 200 µL bioactive ion administration (CS in PBS) by intravenous injection through tail vein every 2 d for 2 weeks while sham and AMI groups received equal volume of PBS. The optimal injection concentrations of “ion therapy” were predetermined by a pre‐experiment, in which two concentrations of CS ion extracts (the CS extracts and 1/2CS dilution) were tested.

*Determination of Myocardial Apoptosis In Vivo*: Mouse myocardial apoptosis was detected by TUNEL by using a commercial kit (catalog 11 684 817 910; Roche Diagnostics). The heart tissues were harvested 28 d after surgery and fixed in 4% paraformaldehyde at room temperature before embedded in paraffin. Heart samples were sectioned to 5 µm along the short axis and transversely across the infarct zone. Sections were then stained according to the manufacturer's protocol. Total nuclei (DAPI staining, blue) and TUNEL positive nuclei (brown‐yellow) in each field were counted in ten randomly chosen fields, and the index of apoptosis (number of TUNEL‐positive nuclei/total number of nuclei × 100%) was calculated.

*Echocardiography*: 28 d after surgery, mice were anesthetized through inhalation of isoflurane (1–1.5%) in O_2_ and echocardiographic examination was performed using a Vevo 2100 System equipped with a 30 MHz transducer (FUJIFILM Visual Sonics, Inc. Toronto, Canada) as previously described^50^ by an observer blinded to the experiment. The LVEDD, LVESD, and the percentage of LV ejection fraction (EF), and FS were detected. All measurements were repeated for at least three consecutive pulsation cycles and the data were averaged for statistical analysis.

*Histological Analysis*: Mice were sacrificed 4 weeks after the induction of myocardial infarction, and hearts samples were collected and fixed in 4% paraformaldehyde overnight and were embedded in paraffin. The weight of hearts samples was measured and the heart weight/body weight (HW/BW) ratio was determined. Samples were sectioned to 5 µm along the short axis, transversely across the infarct zone. Following deparaffinage and dehydration, samples were stained with Masson's trichrome staining was used to detect the collagen deposition in infarct area (collagen was stained blue). The collagen area and LV area was measured by Image‐Pro Plus software (version 6.0; Media Cybernetics, Silver Spring, MD, USA). Fibrosis (%) was calculated as the ratio of collagen area to LV area.

*In Vivo vWF, myh6, Isolectin‐IB4, and Cx43 Immunofluorescence Staining*: In vivo, paraffin sections were prepared as described before. For angiogenesis investigation, paraffin sections were incubated with primary antibodies against von Willebrand factor (vWF, 1:500, Abcam) and then incubated with Alexa Fluor 488 goat antirabbit IgG (Santa Cruz), cell nuclei were stained by DAPI, images of ten randomly selected fields in infarct and border zone were captured by fluorescence microscope, and the blood vessel density is determined as vessels/HPF (high power field) (400×). For capillaries detection, isolectin‐IB4 conjugated with Alexa Fluor 488 (Molecular Probes, Eugene, OR, USA) was used to stain the sections. For gap junction investigation, paraffin sections were incubated with primary antibodies against rabbit anti‐connexin 43 (Abcam, ab11370) and mouse anti‐heavy chain cardiac myosin(Abcam,ab50967) for special labeling of cardiomyocyte. Sections were then incubated with Alexa Fluor 488 goat anti‐rabbit IgG (Santa Cruz) and cy3 goat anti‐mouse IgG (Santa Cruz).

*Plasma NT‐proBNP, VEGFA, AST, ALT and Cr Elisa Assays*: A volume of 600 µL venous blood was collected into tubes containing disodium EDTA, which was centrifuged for 10 min at 3500 rpm. Then the supernatant was collected in EP tube and kept at −80 °C. Plasma NT‐proBNP, VEGFA, AST, ALT, and Cr level were determined by sandwich enzyme‐linked immunosorbent assay (ELISA) with commercially available kits (MSK, KT21109 and KT21108; Mmbio, MM‐44384M2, MM44625M2, and MM‐44455M2, China, respectively) according to the manufacture's instruction. Optical density of each well was determined by using a microplate reader set to 450 nm. The sample values were then read off the standard curve.

*Si Metabolism and Distribution Analysis*: The metabolism of Si was investigated by determining Si concentrations in blood samples collected before and 10, 30, 60, 90, and 120 min after ion injection. The accumulation and distribution of Si in important organs such as heart, lung, liver, and kidney after seven times intravenous injection was also analyzed. The blood and tissue samples were chemically digested in concentrated nitric acid (spectral purity) using a microwave digester and the Si concentrations of the samples were determined by ICP‐MS (PerkinElmer NexION 350X, USA).

*Statistical Analysis*: Data were expressed as mean ± standard deviation. Comparison between two groups was performed with two‐tailed Student's t‐test. Comparisons among more than two groups were performed using one‐way ANOVA followed by post‐hoc Bonferroni test. Significant differences were considered when *p* < 0.05 and *p* < 0.001.

## Conflict of Interest

The authors declare no conflict of interest.

## Supporting information

Supplementary
