# Supplementary material for: Ion Therapy: A Novel Strategy for Acute Myocardial Infarction
Source: Adv Sci (Weinh). 2018 Nov 8;6(1):1801260. doi: 10.1002/advs.201801260 (PMC6325593; doi:10.1002/advs.201801260)
Supplement: Supplementary file 1 — Supplementary [file ADVS-6-1801260-s001.pdf]

Copyright WILEY-VCH Verlag GmbH & Co. KGaA, 69469 Weinheim, Germany, 2018.

ADVANCED SCIENCE
Open Access

Supporting Information

for *Adv. Sci.,* DOI: 10.1002/advs.201801260

Ion Therapy: A Novel Strategy for Acute Myocardial Infarction

Min Yi, Hekai Li, Xiaoya Wang, Jianyun Yan, Long Gao, Yinyan He, Xinglong Zhong, Yanbin Cai, Weijing Feng, Zhanpeng Wen, Chengtie Wu, Caiwen Ou, Jiang Chang,* and Minsheng Chen**

Supporting Information

Table S1 Primers sequences used for Q-RT-PCR.

**Target genes**	**Forward primer sequence (5’ -3’)**	**Reverse primer sequence (5’ -3’)**
Rat Cx43	GAGTCGCATTGGGTGAACATAGA	GTAGACCAGGGAAGCTTACCTG
Human $VEGF_{165}$	TGCGGATCAAACCTCACCA	CAGGGATTTTTCTTGTCTTGCT
Human KDR	GTTCTTTCCACCAGCAGGAAG	TCTAAACCCATGGTGAGACCC
Rat VEGFA	TTCCTGTAGACACACCCACC3	TCCTCCCAACTCAAGTCCAC
Rat cTnT	GGGCTGAACAGCAGCGTAT	CCACTCTTCCGCTCTGTCTG
Rat Myh6	GACAATCTACAGCGGGTGAAGC	TGGCTCGCTGTGTGGTGAA
Rat GAPDH	GGCATCGTGGAAGGGCTCAT	GGGATGACCTTGCCCACAG
Human GAPDH	ACGGATTTGGTCGTATTGGGCG	CTCCTGGAAGATGGTGATGG

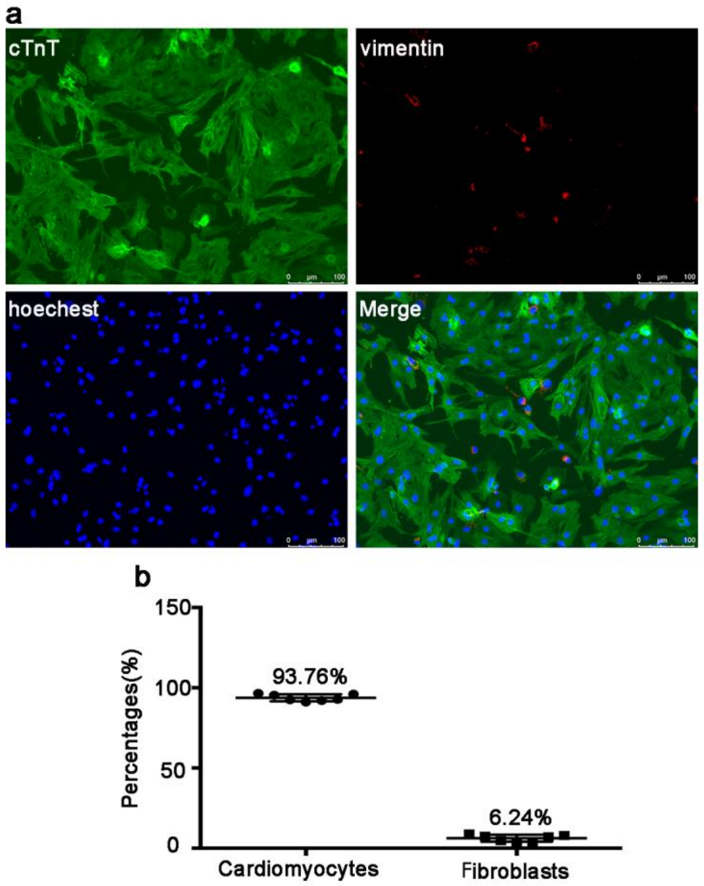

Fig.S1 Percentages of NRCMs in the isolated primary cells. (a) Immunofluorescence images of cTnT (green, cardiomyocyte specific maker) and vimentin (red, fibroblast specific maker) staining. (b) Quantitative analysis of NRCMs percentages in the isolated primary cells (n=5).

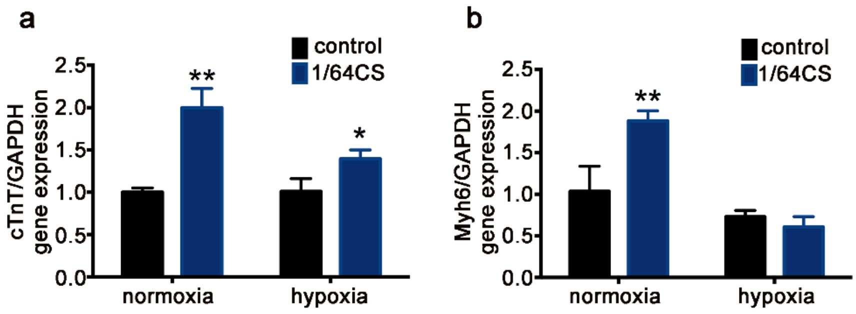

Fig.S2 Effect of silicon-enriched ion extracts on the expression of cardiomyocyte specific marker genes under normoxia and glucose/oxygen deprived conditions in vitro. (a) Expression of cardiomyocyte specific marker gene cTnT under normoxia (cultured for 5 days) and glucose/oxygen deprived conditions (cultured for 120min) in vitro. (b) Expression of cardiomyocyte specific marker gene Myh6 under normoxia (cultured for 5 days) and glucose/oxygen deprived conditions (cultured for 120min) in vitro; *p< 0.05 and

**$p<0.01$ *vs*. control. Data are obtained from three independent experiments; mean ± SD.

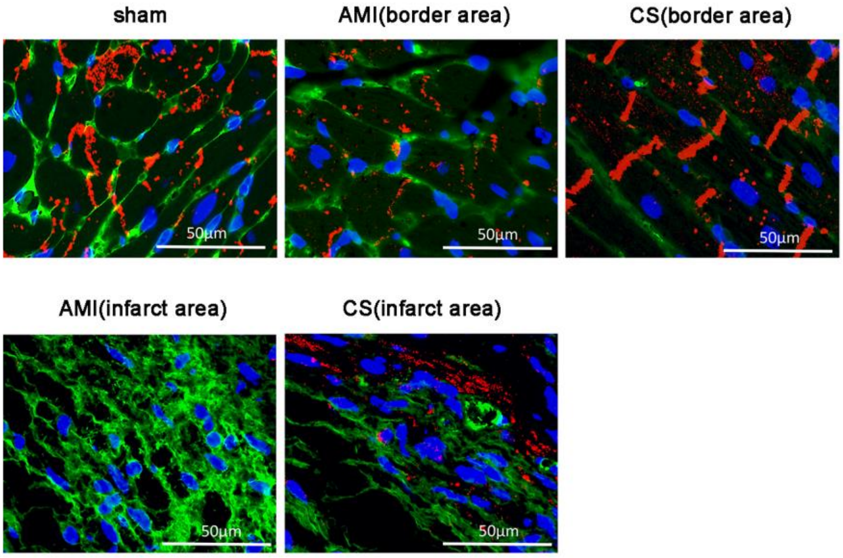

Fig.S3 Gap junction associated Cx43 and Myh6 immunofluorescence staining in cardiomyocytes in vivo. Representative immunofluorescence images of Myh6 (green) and gap junction associated Cx43 (red) staining in the infarcted myocardium 4 weeks after surgery; scale bars represent 50μm.

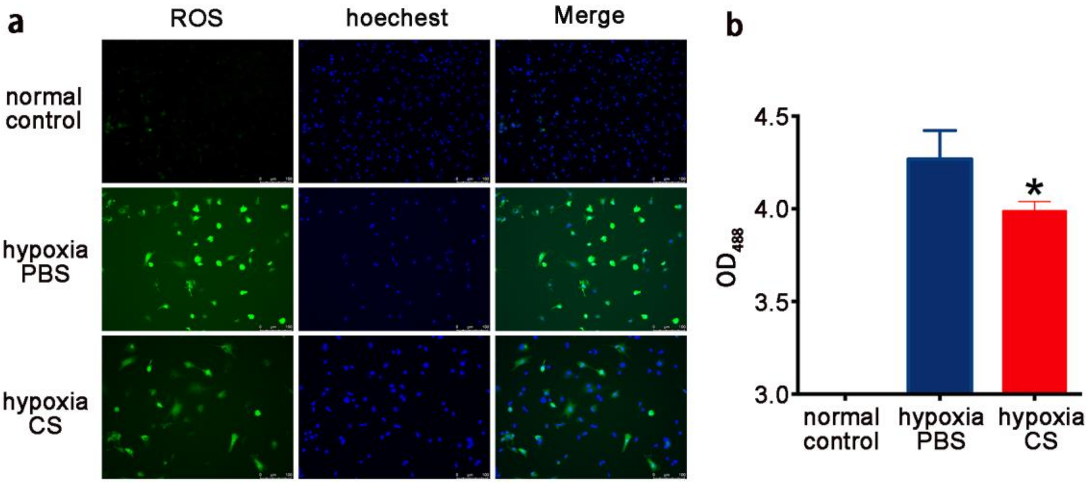

Fig.S4 Effect of silicon-enriched ion extract on ROS levels of NRCMs under normal and glucose/oxygen deprived conditions in vitro. (a) Representative ROS staining examined by DCFH-DA. Normal control means normal oxygen. (b) Quantitative analysis of fluorescence intensity; *$P<0.05$ vs. PBS.

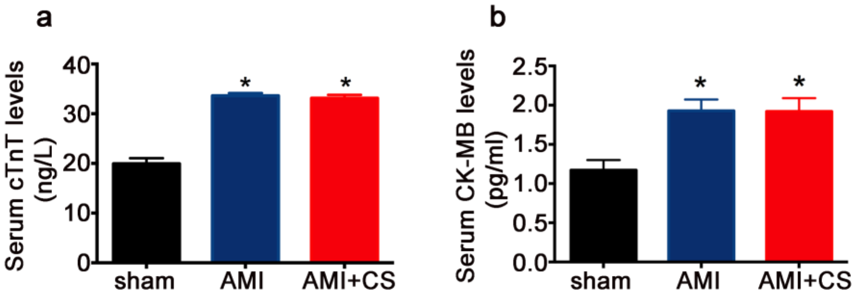

Fig.S5 Cardiac enzymatic expression before "ion therapy" in vivo. (a) Serum expression of cTnT levels in each groups before "ion therapy". (b) Serum expression of CK-MB levels in each groups before "ion therapy". Sham group n=3; AMI group n=6 and AMI+CS group n=6**.** * *p* < 0.05 *vs*. sham group; mean ± SD.

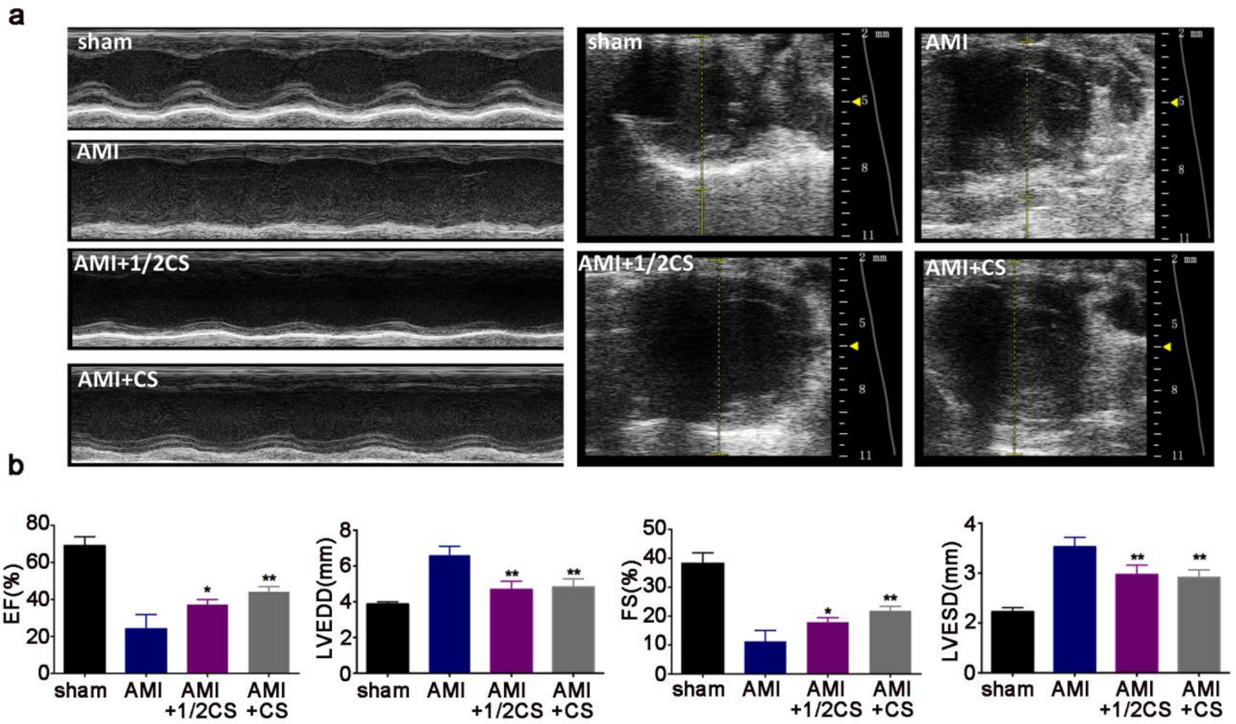

Fig.S6 Effect of "ion therapy" of different concentrations on cardiac function post AMI in vivo. (a) Representative echocardiograms (left) and measurements of different groups (right) were uniformly obtained from the mid-papillary muscle region of the left ventricle (LV) of each groups post "ion therapy" (4 weeks after LAD surgery). (b) Cardiac function measured by left ventricular end-diastolic diameter (LVEDD), LV end-systolic diameter (LVESD), the percentage of LV ejection fraction (EF) and fractional shortening (FS) values. *P < 0.05 *vs*. AMI group, **P < 0.01 vs. AMI group. Sham group n=3;AMI group n=3; AMI+1/2CS1

group n=3 and AMI+ CS group n=3**;** mean ± SD.

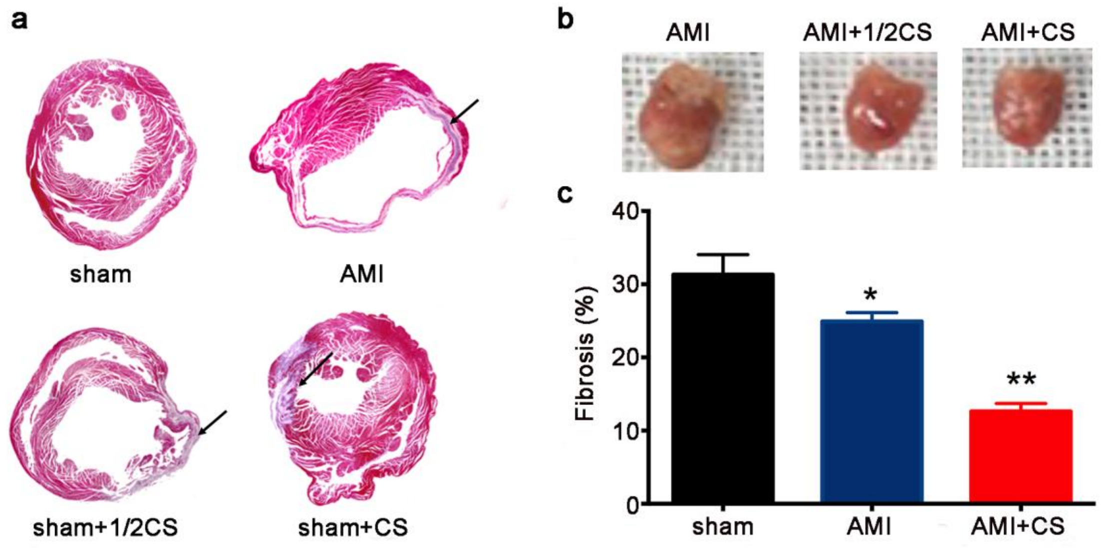

Fig.S7 Effect of "ion therapy" of different concentrations on heart remodeling as well as hypertrophy post AMI in vivo. (a) Representative Masson's trichrome staining of heart sections evaluating collagen deposition 4 weeks after surgery. (c) Quantitative analysis of the area of fibrosis. Sham group was set as 0% and $^{\#}P < 0.001$ *vs.* AMI group (5 pictures for each group). (b) Representative pictures of heart samples indicating hypertrophy degrees. *P < 0.05 *vs*. AMI group, **P < 0.01 vs. AMI group. Sham group n=3;AMI group n=3; AMI+1/2CS1 group n=3 and AMI+ CS group n=3**;** mean ± SD.

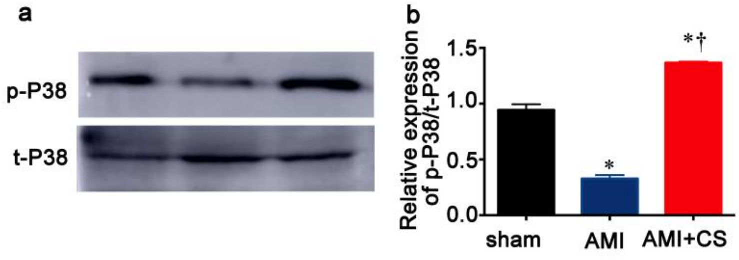

Fig.S8 Effect of "ion therapy" on the expression of P38 protein in the border area of infarcted myocardium. (a) Expression of phosphorylated P38 under "ion therapy" in the border area of infarcted myocardium. (b) Quantification of bands by densitometry. Data are obtained from three independent experiments; *p < 0.01 *vs*. sham, †p < 0.01 *vs*. AMI; mean ± SD.

Text and graphic for Table of Contents

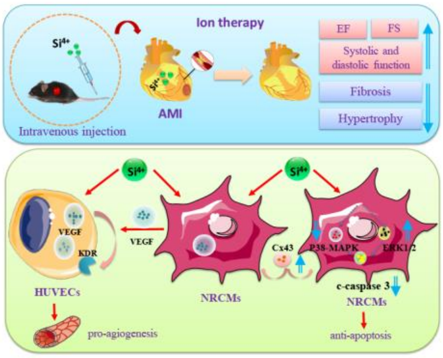

"Ion therapy", a novel strategy for treatment of acute myocardial infarction (AMI) by intravenous injection of bioactive silicon (Si) ions released from silicate bioceramics, can improve cardiac function by promoting cell-cell communication, enhancing gap junction associated Cx43 expression, stimulating VEGF mediated angiogenesis and blood vessel formation, and inhibiting the MAPK family protein-associated apoptosis.
